# Suitability of eastern pines for oviposition and survival of *Sirex noctilio* F.

**DOI:** 10.1371/journal.pone.0174532

**Published:** 2017-03-23

**Authors:** Laurel J. Haavik, Kevin J. Dodds, Jeremy D. Allison

**Affiliations:** 1 Natural Resources Canada, Canadian Forest Service, Great Lakes Forestry Centre, Sault Ste. Marie, ON, Canada; 2 USDA Forest Service, Forest Health Protection, Durham, NH, United States of America; University of Arkansas, UNITED STATES

## Abstract

The European woodwasp, *Sirex noctilio* F., is a pest of pines in many areas around the world. Since its introduction to North America, the distribution of *S*. *noctilio* overlaps with a known host (*Pinus sylvestris*) and hosts native to North America. Direct comparisons of suitability for oviposition and larval survival among these pines have not been made. We tested the relative suitability of four common pine species in northeastern North America (*P*. *sylvestris*, *P*. *resinosa*, *P*. *banksiana*, and *P*. *strobus*) as hosts for *S*. *noctilio* in a controlled, but *in situ* experiment. In a mixed pine forest in northern Ontario, we caged *S*. *noctilio* mating pairs on 10 freshly cut pine logs of each species, and estimated oviposition, counted adult *S*. *noctilio* (F1 generation) that emerged from logs, and calculated survivorship from egg to adult. *Pinus sylvestris* and *P*. *resinosa* were optimal hosts according to all three metrics of *S*. *noctilio* performance. *Pinus strobus* was a suitable larval host, but was not perceived as such by females, as evidenced by lower oviposition. *Pinus banksiana* was perceived as a suitable host by females, but was the least suitable larval host. Our results suggest that *P*. *sylvestris* and *P*. *resinosa* are more suitable hosts, at least in cut logs, than *P*. *strobus* and *P*. *banksiana* for *S*. *noctilio* in eastern North America.

## Introduction

The European woodwasp, *Sirex noctilio* F., is a wood-boring pest of pines (*Pinus*) in many, but not all, parts of the world where it has been introduced [1]. *Sirex noctilio* is especially aggressive in areas of the Southern Hemisphere where natural enemies and other wood-boring competitors are absent and exotic pines are planted in monocultures that are not well-managed [1, 2]. Though it has not been a destructive pest in North America since its discovery in 2004 [3, 4], *S*. *noctilio* has potential to impact pine forests of varying species compositions differently as it spreads. In North America, the range of *S*. *noctilio* overlaps with *P*. *sylvestris* (Scots pine), *P*. *resinosa* (red pine), *P*. *banksiana* (jack pine), and *P*. *strobus* (eastern white pine) [5–7]. *Sirex noctilio* shares a co-evolutionary history with *P*. *sylvestris* (both are native to Europe), which is widely planted and considered naturalized in eastern North America [8]. The other three pines are native to North America and do not share a co-evolutionary history with *S*. *noctilio*. Understanding the relative suitability of these pines for *S*. *noctilio* oviposition and larval development is critical for predicting whether and where the woodwasp could become an aggressive pest in North America as it spreads.

Offspring (brood) success in endophytic insects is determined by a combination of perceived suitability of the larval host by the female and actual suitability of the host habitat for larval development [9–11]. Volatiles (α- and β-pinene, carene, and others) emitted by stressed pines attract female *S*. *noctilio* [12–14]. The female first assesses the bark with her antennae, then probes and eventually drills into the bark with her ovipositor [15]. She determines the suitability of the tree for larval development based on osmotic (resin) pressure in the phloem, and will not oviposit in trees with high osmotic pressure [16–18]. If osmotic pressure is high, she may still inject a fungal symbiont, usually *Amylostereum areolatum*, and a phytotoxic venom that function in combination to weaken the tree’s vascular system [16–20]. If osmotic pressure is low, the female considers the tree a suitable host, and she will also inject one or more eggs in or near her initial drill site [16–18].

Less is known about what constitutes a suitable larval host. Tree resistance likely plays a primary role in dictating whether small larvae survive, and was the most important mortality factor during *S*. *noctilio* development according to life tables [21]. Pines resist *S*. *noctilio* constitutively by flooding oviposition drills with resin [22], and by induced accumulation of polyphenols in the sapwood surrounding the oviposition site [22, 23]. Both of those mechanisms are likely compromised in stressed trees. It is not known how host tree condition relates to nutritional quality for developing larvae, partly because the source(s) of larval nutrition is(are) unclear. Larvae chew wood, but pass it underneath the body instead of through the digestive tract, and may feed on liquid extract digested first by the fungus to release starch and sugars [24]. Bacteria may also provide nutrition for *S*. *noctilio* larvae, either through cellulose digestion, provision of sterols, and/or nitrogen fixation [24–26].

Despite unknowns surrounding host suitability for *S*. *noctilio* larvae, there are many host reports from around the world. *Sirex noctilio* attacks and develops in many different pines, and while some species seem to be preferred hosts, or at least more suitable hosts than others, no pines appear to be resistant [27]. This suggests that *S*. *noctilio* and its fungal symbiont are well-adapted to develop in all species of the Pinaceae. Fir (*Abies*) and spruce (*Picea*) are occasional hosts for *S*. *noctilio* in its native range, though far less frequently than pines [15, 28]. In Europe, *S*. *noctilio* colonizes the native pines, *P*. *pinaster* and *P*. *sylvestris* more frequently than *P*. *radiata* (not native to Europe) [28–30]. In the Southern Hemisphere, *S*. *noctilio* commonly colonizes *P*. *radiata*, *P*. *taeda*, *P*. *elliotti*, *P*. *ponderosa*, and *P*. *patula* [1, 31–33].

In North America, observational studies reported that *P*. *sylvestris* is likely a more favorable and/or suitable host than other pines. *Sirex noctilio* attacks and kills more trees in *P*. *sylvestris* than in *P*. *resinosa* forests [5, 29]. In naturally infested trees collected from different sites, larger broods of *S*. *noctilio* and its parasitoids were observed in *P*. *sylvestris* than in *P*. *resinosa*, *P*. *banksiana*, or *P*. *strobus* [6, 7, 34, 35], although none of those studies compared all four pine species at once. In a study where co-occurring *P*. *sylvestris* and *P*. *resinosa* were artificially stressed with herbicides, *S*. *noctilio* brood from *P*. *sylvestris* were 25% larger in densities three times greater than brood from *P*. *resinosa* [36]. Direct comparisons of the suitability of different pines in controlled settings have not been made.

Our objective was to test the relative suitability of four common pine species in northeastern North America (*P*. *sylvestris*, *P*. *resinosa*, *P*. *banksiana*, and *P*. *strobus*) as hosts for *S*. *noctilio* in a controlled, but *in situ* experiment. We tested whether pine species affected *S*. *noctilio* (1) oviposition, (2) F1 brood production, and (3) survivorship, as well as (4) abundance of parasitoids. Based on observations from previous studies, we predicted that *S*. *noctilio* oviposition, F1 brood production, and survivorship would all be greater in *P*. *sylvestris* than in the other pines.

## Materials and methods

### Study site and adult *S*. *noctilio* collection

The study site was a mixed pine-hardwood (maple, birch) forest (UTM Zone 16T, 692978 E, 5152387 N) near Sault Ste. Marie, Ontario, Canada, where pole-sized pines of each species grew. No specific permission was required to use this study site, as the land was owned by the Canadian Forest Service. We found evidence of *Sirex* at the study site, via resin beading [37] on the trunks of a few dying *P*. *sylvestris*. This resin beading could have been from *S*. *noctilio*, or the closely related native species, *S*. *nigricornis*. Although *S*. *noctilio* was not confirmed as present in the area at the time this study began (July 2014), *S*. *nigricornis* was and because it has the same parasitoid complex as *S*. *noctilio* in eastern North America [38], we expected that the parasitoids, *Ibalia leucospoides* (Hymenoptera: Ibaliidae) and *Rhyssa persuasoria* and *R*. *lineolata* (Hymenoptera: Ichneumonidae), would be present at the study site, as well as competing wood borers, *Monochamus* spp. (Coleoptera: Cerambycidae).

We introduced adult *S*. *noctilio* to freshly cut pine logs, enclosed in cages at our study site (see *Experimental set-up* below). These adult *S*. *noctilio* were obtained from naturally infested pines (identified by resin beading), which we felled and removed from several sites throughout southern Ontario (see [39] for details on locations). In late June of 2014, we brought infested pine logs to a field station near Barrie, Ontario, where we placed them into cardboard rearing tubes in a covered, outdoor shed. We collected *S*. *noctilio* that emerged from the logs 5x per week and stored them at 4°C until enough were available for a shipment (20+ wasps). We shipped the wasps to Sault Ste. Marie overnight in coolers with ice packs (1–2 times per week).

### Experimental set-up

We selected 10 pole-sized pines (diameter at breast height: 6–8 cm) of each species. We felled five trees of each species during the first week of July, and five more during the second week of July (to prepare for staggered availability of adult *S*. *noctilio*). As described in Haavik et al. [21], we cut a 3–4 m section from the mid-bole of each tree and placed each end of these logs onto a cinder block (25x10x10 cm). On the same day, we then secured two wire mesh (2x2 mm) screen cages (1 m long) to each horizontal log with heavy duty, plastic zip-ties at each end. The cages were equipped with Velcro® closures, sewn directly onto the wire mesh. To prevent cages from collapsing in on themselves, we screwed three wooden struts (10–15 cm long, cut from 5x5 cm lumber) into logs, equidistant apart (e.g., at 0°, 120°, and 240°), near both ends of cages, and encircled the struts in a hoop of plastic tubing (1.75 cm in diameter), screwed to the struts. The logs were arranged in a randomized complete block design in a clearing within the mixed pine-hardwood forest.

In an attempt to create a physically suitable (i.e., stressed) host tree for optimal survival of the *S*. *noctilio* F1 generation (see [40]), we felled and caged trees 1.5–3 weeks before introducing *S*. *noctilio* mating pairs to cages. Each cage received two male-female pairs of *S*. *noctilio* (i.e., 4 wasps per cage, 8 wasps per tree, 80 wasps per treatment, 320 wasps total [160 males, 160 females]). To expose one-half of each log to associates (competitors + parasitoids) (n = 10 logs per pine species), we removed one cage from each log (top or bottom section decided at random by a coin flip) 1–2 weeks after wasps were inserted into cages. At this time all females were dead, and had presumably oviposited on logs. The remaining bole sections were protected from associates, and cages were left on logs (n = 10 logs per pine species) throughout development of the F1 generation to protect them from associates.

In late June of 2015, just prior to expected completion of the F1 generation, we removed logs from the field site, and returned them to rearing drums in the lab. We collected emerging adult wasps 2–5 times per week through October 2015. After adult emergence was complete, we peeled the bark from logs with a drawknife to reveal the sapwood surface. To estimate the number of eggs oviposited by female *S*. *noctilio* into the wood, we used a dissecting microscope (6.4x), positioned over the log surface, to count the number and type (single, double, triple, etc.) of oviposition drills (see [16] for details on this method).

### Data analysis

We used an equation developed by Madden [16], which we tested and used previously [21], to estimate the number of eggs oviposited by female *S*. *noctilio* (i.e., realized fecundity) based on oviposition drill type (Total no. eggs = 0.04*no. single drills + 0.68*no. double drills + 1.55*no. triple drills + 2.22*no. quadruple drills).

In the R statistical environment [41], we examined histograms of response variables to determine appropriate models and error terms. All were nested models with tree as a random factor and cage (exposed or protected from associates) and pine species as fixed factors. Interaction terms were not significant, so we removed them from models. The effects of cage and pine species on number of eggs was tested with a generalized linear model with a negative binomial distribution of errors (package = MASS), and differences among species were investigated with least squares means. We used a hurdle model with a negative binomial distribution of errors (package = pscl) to test for the effects of cage and pine species on number of *S*. *noctilio* adults. Relative to generalized linear models, hurdle models are advantageous because zero and non-zero responses are tested separately [42]. Hurdle models determine if the frequency of zero responses differs more than by chance among the treatments [42]. The effects of cage and pine species on survivorship from egg to adult (only trees in which eggs were oviposited were tested) was tested with a generalized linear model with a binomial distribution of errors (package = stats), and differences among species were investigated with least squares means. Too few parasitoids and competitors were collected from logs for statistical analysis. Statistical significance was set at α = 0.05.

## Results

*Sirex noctilio* oviposition occurred in 62 of the 80 logs in the entire study. We estimated that a total of 2,661 eggs were oviposited in the logs by *S*. *noctilio*; 732 in *P*. *banksiana*; 710 in *P*. *resinosa*; 1,110 in *P*. *sylvestris*; and 110 in *P*. *strobus*. In total, 145 *S*. *noctilio* adults (F1 generation) were recovered from the logs (overall survivorship from egg to adult = 5%); 6 from *P*. *banksiana*; 53 from *P*. *resinosa*; 64 from *P*. *sylvestris*; and 22 from *P*. *strobus*.

**Fig 1:**
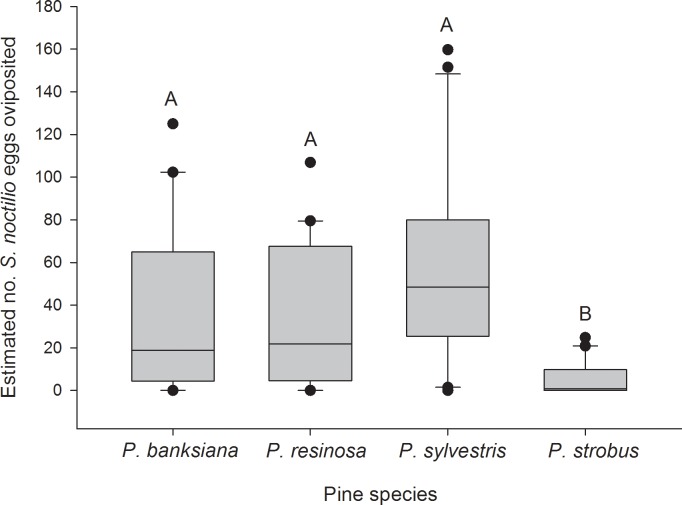
Boxplots of estimated number of eggs oviposited by *Sirex noctilio* in each pine species. n = 20 logs per *Pinus* species. Boxes are bounded by the first and third quartiles; the internal solid line represents the median.

**Fig 2:**
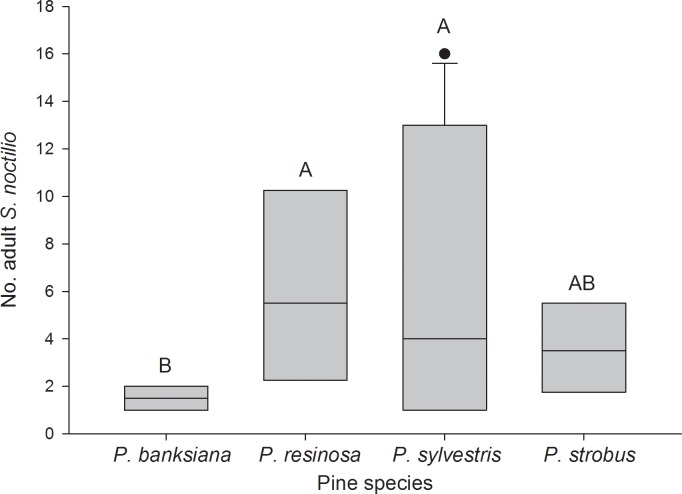
Boxplots of number of adult *Sirex noctilio* that emerged from each pine species. n = 4 logs for *Pinus banksiana*; n = 8 logs for *P*. *resinosa*; n = 11 logs for *P*. *sylvestris*; n = 6 logs for *P*. *strobus*. Logs that did not produce *S*. *noctilio* adults were excluded. Boxes are bounded by the first and third quartiles; the internal solid line represents the median.

**Fig 3:**
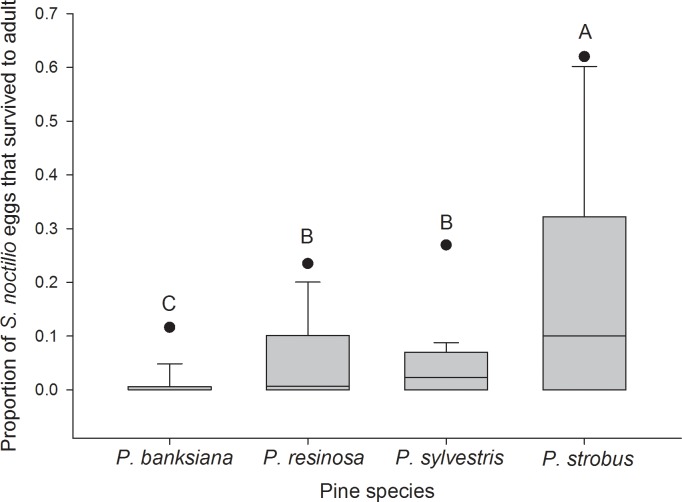
Boxplots of the proportion of *Sirex noctilio* eggs that survived to adulthood in each pine species. n = 16 logs for *Pinus banksiana* and *P*. *resinosa*; n = 19 logs for *P*. *sylvestris*; n = 10 logs for *P*. *strobus*. Boxes are bounded by the first and third quartiles; the internal solid line represents the median.

Significantly fewer *S*. *noctilio* eggs were oviposited in *P*. *strobus* compared with the other species (z = 4.16; df = 3,76; *P* < 0.001), which did not differ significantly from one another (Fig 1). Among logs that produced *S*. *noctilio* brood adults (29 of 80), the number of *S*. *noctilio* produced by *P*. *sylvestris* and *P*. *resinosa* were greater than the number produced by *P*. *banksiana* (z = 2.55; df = 9,19; *P* = 0.012), and the number produced by *P*. *strobus* did not differ significantly from the other pine species (Fig 2). The number of logs that did not produce adult *S*. *noctilio* (51 of 80) was greater for *P*. *banksiana* (16) than for *P*. *sylvestris* (9) (z = 2.21; df = 9,41; *P* = 0.027); *P*. *resinosa* (12) and *P*. *strobus* (14) did not differ significantly from the others. Survival from egg to adult was greatest in *P*. *strobus* (z = 4.10; df = 3,58; *P* < 0.001), intermediate in *P*. *sylvestris* and *P*. *resinosa* (z = 4.65 and 5.25, respectively; df = 3,58; *P* < 0.001), and lowest in *P*. *banksiana* (z = 4.10; df = 3,58; *P* < 0.001) (Fig 3).

The effect of exclusion cage on estimated number of *S*. *noctilio* eggs, number of brood adults, or survivorship was not significant in any of the models (*P* > 0.05 for all). No *Rhyssa* individuals were recovered from the exposed logs. Few *Ibalia leucospoides* emerged from any of the exposed logs (14 in total). No *I*. *leucospoides* emerged from *P*. *banksiana*; one emerged from a *P*. *strobus* log; one, two, and three emerged from three different *P*. *resinosa* logs; one, three, and three emerged from three different *P*. *sylvestris* logs. No Cerambycidae individuals were recovered from the logs. A few bark beetles (Scolytinae) were recovered from the exposed logs, but were not counted. We expected that at least some associates had already exited logs by late June of 2015 (when logs were removed from the field site).

## Discussion

*Pinus sylvestris* and *P*. *resinosa* were equally suitable hosts for *S*. *noctilio*, both in perception by females and in brood production and survivorship from egg to adult. *Pinus strobus* was a suitable larval host, but was not perceived as such by females. *Pinus banksiana* was perceived as a suitable host by females, but was the least suitable larval host of all pine species (smallest brood, lowest survivorship, and most logs with no brood). Survivorship from egg to adult was very low among all pine species (5%), much lower than that reported by Taylor [43] in *P*. *radiata* (36–71%). Those studies measured survival in naturally attacked pines, which female *S*. *noctilio* selected as suitable larval hosts, whereas our study forced that interaction by not providing females with a choice of hosts of varying condition. In our previous study that used the same method, survivorship was also low (1–14%) [21]. It is likely that in felling trees 1.5–3 weeks before *S*. *noctilio* oviposition we created controlled experimental conditions, but failed to artificially create optimal hosts for larval development.

Our results relate to the preference-performance hypothesis (PPH, also called the mother-knows-best hypothesis) [9, 10]. The PPH predicts that females will choose to lay eggs on the host plant that is optimal for larval survival and development [9, 10]. Though *S*. *noctilio* females were not presented with a choice of larval hosts in our study, which precluded a direct test of the PPH, their behavior was consistent with the PPH only some of the time. Females apparently chose not to oviposit at all in some cases, especially on *P*. *strobus*, which turned out to be a good host for larvae. In contrast, *S*. *noctilio* females were apparently not able to detect that *P*. *banksiana* would not be an optimal larval host.

With a few exceptions [11, 44], the PPH theory was supported in a majority of empirical studies, compared via meta-analysis [44]. Specifically, Gripenberg et al. [44] found that in many insects, females laid more eggs on plants that were the most suitable for offspring, and more offspring survived on these preferred hosts. The PPH was especially well supported among insects that were oligophagous, having an intermediate host range, compared with insects that were polyphagous or monophagous [44]. The PPH was supported among oligophagous insects that, like *S*. *noctilio*, live and feed as larvae in woody tissues, such as the emerald ash borer (*Agrilus planipennis*) [45], the eucalyptus longhorned borer (*Phorocantha semipunctata*) [46], and the brown spruce longhorn beetle (*Tetropium fuscum*, stressed vs. healthy hosts of same species) [47]. In a polyphagous wood-boring insect, the Asian longhorned beetle (*Anoplophora glabripennis*), females only sometimes chose the host that also conferred optimal larval survival [48]. Although *S*. *noctilio* is oligophagous, based on our results, we predict that females would not consistently choose optimal hosts for larval survival and development, if given a choice.

Perhaps host acceptance cues varied among the different pine species. Böröczky et al. [14] found that a *P*. *sylvestris* chemotype with high amounts of the volatile carene was more attractive to female *S*. *noctilio* than *P*. *strobus* and a *P*. *sylvestris* chemotype with less carene, which suggests that host volatiles could be important in oviposition decisions. This highlights the need for a better understanding of the complete chemical volatile profiles of eastern pines and their relative attractiveness to *S*. *noctilio* females. Blends of α/β-pinene [49] or ethanol + α-pinene [50] were especially attractive to Siricidae native to North America. Bark texture may also be an important host cue. Other wood-boring insects that lay eggs on bark often prefer rough, or cracked bark over smooth bark [51, 52], presumably because bark fissures provide protection from natural enemies and environmental extremes, or provide a tight spot in which newly hatched larvae can attain leverage to chew and burrow into the bark. *Sirex noctilio* females have a different oviposition strategy, and drill past the bark to lay eggs directly in the sapwood. Still, smooth bark, such as that on *P*. *strobus*, may interfere with the female’s purchase during drilling; or, perhaps more likely, smooth bark may be more accessible to parasitoids, especially those with short ovipositors, like *I*. *leucospoides*.

There are very few empirical examples that support the idea that female herbivorous insects seek enemy-free space for oviposition [10, 53]. Female *S*. *noctilio* avoidance of smooth-barked *P*. *strobus* may be an adaptation to avoid parasitoids. Unfortunately, the environment in our study site did not appear to support a very sizable parasitoid population to test this, as evidenced by the fact that *S*. *noctilio* survival was not affected by protection from natural enemies. Further, *I*. *leucospoides* attraction to and oviposition on different pine species may be an indirect response influenced by density of *S*. *noctilio* larvae present in a tree rather than the species of tree. Studies show that *I*. *leucospoides* is an efficient forager that can discern abundance of *S*. *noctilio* larvae in pine logs from a distance [54, 55].

## Conclusions

Among the four potential host species evaluated, *P*. *sylvestris* and *P*. *resinosa* were optimal hosts for *S*. *noctilio* larval survivorship. We found little difference between *P*. *sylvestris* and *P*. *resinosa* as hosts for *S*. *noctilio*. This conflicts with observational studies that suggested *P*. *sylvestris* is a better host for *S*. *noctilio* than *P*. *resinosa*, *P*. *banksiana*, and *P*. *strobus*. There may be differences in attraction and suitability of living trees (those studies) versus cut logs (our study). In addition, those studies compared the relative number of *S*. *noctilio* brood adults produced by the different pine species that were colonized by ambient populations of *S*. *noctilio* [6, 7, 34, 36], or the relative number of different pine species that were killed by *S*. *noctilio* in different stands [5, 29]. In those studies, it is likely that the local population density of *S*. *noctilio*, host condition, and availability of suitable hosts all played a larger role than actual differences in suitability of the two pine species as hosts for *S*. *noctilio* larvae. For example, in eastern North America, *P*. *resinosa* forests are more economically important and more heavily managed than *P*. *sylvestris* forests, and would be less likely to have as many hosts in a stressed condition suitable for *S*. *noctilio* survival and population growth.

## Supporting information

S1 Table Data collected from each log sample. Data includes number of *Sirex noctilio* eggs oviposited by females in the parent generation (estimate; see text for details), and number of adult S. noctilio in the F2 generation.
(DOCX)

## References

[1] Hurley BP, Slippers B, Wingfield MJ. A comparison of control results for the alien invasive woodwasp, *Sirex noctilio*, in the southern hemisphere. Agric For Entomol. 2007;9:159–71.

[2] Rawlings GB. Recent observations on the *Sirex noctilio* population in *Pinus radiata* forests in New Zealand. NZ J For. 1948;5:411–21.

[3] Hoebeke ER, Haugen DA, Haack RA. *Sirex noctilio*: discovery of a palearctic siricid woodwasp in New York. Newsletter of the Michigan Entomological Society. 2005;50:24–5.

[4] de Groot P, Nystrom K, Scarr T. Discovery of *Sirex noctilio* (Hymenoptera: Siricidae) in Ontario, Canada. The Great Lakes Entomologist. 2006;39:49–53.

[5] Dodds KJ, de Groot P, Orwig D. The impact of *Sirex noctilio* in *Pinus resinosa* and *Pinus sylvestris* stands in New York and Ontario. Can J For Res. 2010;40:212–23.

[6] Ryan K, De Groot P, Nott RW, Drabble S, Ochoa I, Davis C, et al Natural enemies associated with *Sirex noctilio* (Hymenoptera: Siricidae) and *S*. *nigricornis* in Ontario, Canada. Environ Entomol. 2012;41:289–97. 10.1603/EN11275 22507001

[7] Zylstra KE, Mastro VC. Common mortality factors of woodwasp larvae in three northeastern United States host species. Journal of Insect Science. 2012;12:1–8. 10.1673/031.012.8301 PMC3596941 23421560

[8] Skilling DD. Scotch pine, Pinus sylvestris L. Silvics of North America. 1: USDA Forest Service Agriculture Handbook 654;1990. p. 1383.

[9] Jaenike J. On optimal oviposition behavior in phytophagous insects. Theor Popul Biol. 1978;14:350–6. 751265 10.1016/0040-5809(78)90012-6

[10] Thomson JN. Evolutionary ecology of the relationshp between oviposition preference and performance of offspring in phytophagous insects. Entomol Exp Appl. 1988;47:3–14.

[11] Mayhew PJ. Adaptive patterns of host-plant selection by phytophagous insects. Oikos. 1997; 79:417–28.

[12] Simpson RF, McQuilkin RM. Identification of the volatiles from felled *Pinus radiata* and the electroantennograms they elicit from *Sirex noctilio*. Entomol Exp Appl. 1976;19:205–13.

[13] Simpson RF. Bioassay of pine oil components as attractants for *Sirex noctilio* (Hymenoptera: Siricidae) using electroantennogram techniques. Entomol Exp Appl. 1976; 19:11–8.

[14] Böröczky K, Zylstra KE, McCartney NB, Mastro VC, Tumlinson JH,. Volatile profile differences and the associated *Sirex noctilio* activity in two host tree species in the Northeastern United States. J Chem Ecol. 2012;38:213–21. 10.1007/s10886-012-0077-y 22359190

[15] Madden JL. *Sirex* in Australasia In: Berryman AA, editor. Dynamics of Forest Insect Populations. New York: Plenium Pub. Corp.;1988 p. 407–29.

[16] Madden JL. Oviposition behavior of the woodwasp, *Sirex noctilio* F. Aust J Zool. 1974;22:341–51.

[17] Madden JL, Coutts MP. The role of fungi in the biology and ecology of woodwasps (Hymenoptera: Siricidae) In: Batra LR, editor. Insect-fungus symbiosis: nutrition, mutualism and commensalism. Montclair: John Wiley & Sons Inc.;1979 p. 288.

[18] Coutts MP, Dolezal JE. Emplacement of fungal spores by the woodwasp, *Sirex noctilio*, during oviposition. For Sci. 1969;15:412–6.

[19] Coutts MP. The formation of polyphenols in small blocks of *Pinus radiata* sapwood with and without the fungal symbiont of *Sirex*. Aust For Res. 1969;4:29–34.

[20] Coutts MP. The mechanism of pathogenicity of *Sirex noctilio* on *Pinus radiata* I. The effects of the symbiotic fungus *Amylostereum* Spp. (Thelophoraceae). Aust J Biol Sci. 1969;22:915–24.

[21] Haavik LJ, Dodds KJ, Allison JD. Do native insects and associated fungi limit non-native woodwasp, *Sirex noctilio*, survival in a newly invaded environment? PLoS ONE. 2015; 10:e0138516 10.1371/journal.pone.0138516 26447845 PMC4598122

[22] Coutts MP, Dolezal JE. Polyphenols and resin in the resistance mechanims of *Pinus radiata* attacked by the wood wasp, *Sirex noctilio*, and its associated fungus Commonwealth of Australia, Department of National Development Forestry and Timber Bureau 1966;Leaflet No. 101:14.

[23] Hillis WE, Inoue T. The formation of polyphenols in trees—IV. The polyphenols formed in *Pinus radiata* after *Sirex* attack. Phytochem. 1968;7:13–22.

[24] Thompson BM, Bodart J, McEwen C, Gruner DS. Adaptations for symbiont-mediated external digestion in *Sirex noctilio* (Hymenoptera: Siricidae). Ann Entomol Soc Am. 2014;107:453–60.

[25] Adams AS, Jordan MS, Adams SM, Suen G, Goodwin LA, Davenport KW, et al Cellulose-degrading bacteria associated with the invasive woodwasp *Sirex noctilio*. The ISME Journal. 2011;5:1323–31. 10.1038/ismej.2011.14 21368904 PMC3146269

[26] Takasuka TE, Book AJ, Lewin GR, Currie CR, Fox BG. Aerobic deconstruction of cellulosic biomass by an insect-associated *Streptomyces*. Scientific Reports. 2013;3:10 pp. 10.1038/srep01030 PMC3538285 23301151

[27] Ryan K, Hurley B. Life history and biology of *Sirex noctilio* In: Slippers B, de Groot P, Wingfield MJ, editors. The Sirex woodwasp and its fungal symbiont: research and management of a worldwide invasive pest. New York: Springer;2012 p. 15–30.

[28] Spradbery JP, Kirk AA. Aspects of the ecology of siricid woodwasps (Hymenoptera: Siricidae) in Europe, North Africa and Turkey with special reference to the biological control of *Sirex noctilio* F. in Australia. Bull Ent Res. 1978;68:341–59.

[29] Ayres MP, Pena R, Lombardo JA, Lombardero MJ. Host use patterns by the European woodwasp, *Sirex noctilio*, in its native and invaded range. PLoS ONE. 2014;9:e90321 10.1371/journal.pone.0090321 24675574 PMC3968001

[30] Lombardero MJ, Ayres MP, Krivak-Tetley FE, Fitza KNE. Population biology of European woodwasp, *Sirex noctilio*, in Galica, Spain. Bulletin Ent Res. 2016; 106:569–80. 10.1017/S0007485316000043 26907681

[31] Rawlings GB, Wilson NM. *Sirex noctilio* as a beneficial and destructive insect to *Pinus radiata* in New Zealand. NZ J For. 1949;6:20–9.

[32] Carnegie AJ, Matsuki M, Haugen DA, Hurley BP, Ahumada R, Klasmer P, et al Predicting the potential distribution of *Sirex noctilio* (Hymenoptera: Siricidae), a significant exotic pest of *Pinus* plantations. Ann For Sci. 2006;63:119–28.

[33] Nahrung HF, Ramsden M, Griffiths M. Sirex woodwasp range expansion in Australia: performance and parasitism on two commercial pine species. Forestry. 2016;89:310–5.

[34] Eager PT, Allen DC, Frair JL, Fierke MK. Within-tree distributions of the *Sirex noctilio* Fabricius (Hymenoptera: Siricidae)—parasitoid complex and development of an optimal sampling scheme. Environ Entomol. 2011;40:1266–75. 10.1603/EN10322 22251737

[35] Foelker CJ, Standley CR, Parry D, Fierke MK. Complex ecological relationships among an assemblage of indigenous hymenopteran parasitoids, the exotic European woodwasp (*Sirex noctilio*; Hymenoptera: Siricidae), and a native congener. Can Entomol. 2016;148:532–42.

[36] Foelker CJ. Beneath the bark: associations among *Sirex noctilio* development, bluestain fungi, and pine host species in North America. Ecol Entomol. 2016;41:676–84.

[37] Ryan K, de Groot P, Smith SM, Turgeon JJ. Seasonal occurrence and spatial distribution of resinosis, a symptom of *Sirex noctilio* (Hymenoptera: Siricidae) injury, on boles of *Pinus sylvestris* (Pinaceae). Can Entomol. 2013;145:117–22.

[38] Coyle DR, Gandhi KJK. The ecology, behavior, and biological control potential of hymenopteran parasitoids of woodwasps (Hymenoptera: Siricidae) in North America. Environ Entomol. 2012; 41:731–49.

[39] Haavik LJ, Dodds KJ, Ryan K, Allison JD. Evidence that the availability of suitable pine limits non-native *Sirex noctilio* in Ontario. Agric For Entomol. 2016;18:357–66.

[40] Madden JL. Some treatments which render Monterey pine (*Pinus radiata*) attractive to the wood wasp *Sirex noctilio* F. Bull Ent Res. 1971;60:467–72.

[41] R: A language and environment for statistical computing ISBN 3-900051-07-0, URL http://www.R-project.org ed. Vienna, Austria: R Foundation for Statistical Computing; R development Core Team 2015.

[42] Zeileis A, Kleiber C, Jackman S. Regression models for count data in R. Journal of Statistical Software. 2008;27.

[43] Taylor KL. Evaluation of the insect parasitoids of *Sirex noctilio* (Hymenoptera: Siricidae) in Tasmania. Oecologia. 1978;32:1–10. 28308663 10.1007/BF00344686

[44] Gripenberg S, Mayhew PJ, Parnell M, Roslin T. A meta-analysis of preference-performance relationships in phytophagous insects. Ecology Letters. 2010;13:383–93. 10.1111/j.1461-0248.2009.01433.x 20100245

[45] Rigsby CM, Muilenburg VL, Tarpey T, Herms DA, Cipollini D. Oviposition preferences of *Agrilus planipennis (*Coleoptera: Buprestidae) for different ash species support the mother knows best hypothesis. Ann Entomol Soc Am. 2014;107:773–81.

[46] Hanks LM, Paine TD, Millar JG. Host species preference and larval performance in the wood-boring beetle *Phoracantha semipunctata* F. Oecologia. 1993;95:22–9. 28313307 10.1007/BF00649502

[47] Flaherty L, Quiring DT, Pureswaran D, Sweeney J. Preference of an exotic wood borer for stressed trees is more attributable to pre-alighting than post-alighting behaviour. Ecol Entomol. 2013;38:546–52.

[48] Morewood WD, Neiner PR, McNeil JR, Sellmer JC, Hoover K. Oviposition preference and larval performance of *Anoplophora glabripennis* (Coleoptera: Cerambycidae) in four eastern North American hardwood tree species. Environ Entomol. 2003;32:1028–34.

[49] Coyle DR, Pfammatter JA, Journey AM, Pahs TL, Cervenka VJ, Koch RL. Community composition and phenology of native Siricidae (Hymenoptera) attracted to semiochemicals in Minnesota. Environ Entomol. 2012;41:91–7. 10.1603/EN11192 22525063

[50] Erbilgin N, Stein JD, Acciavatti RE, Gillette NE, Mori SR, Bischel K, et al A blend of ethanol and (α-pinene were highly attractive to native siricid woodwasps (Siricidae, Siricinae) infesting conifers of the Sierra Nevada and the Allegheny Mountains. J Chem Ecol. 2017;in press. 10.1007/s10886-016-0803-y 28032268

[51] Donley DE. Oviposition by the red oak borer, *Enaphalodes rufulus* Coleoptera: Cerambycidae. Ann Entomol Soc Am. 1978;71:496–8.

[52] Marshall JM, Smith EL, Mech R, Storer AJ. Estimates of *Agrilus planipennis* infestation rates and potential survival of ash. Am Midl Nat. 2013;169:179–93.

[53] Björkman C, Larsson S, Bommarco R. Oviposition preferences in pine sawflies: a trade-off between larval growth and defence against natural enemies. Oikos. 1997;79:45–52.

[54] Corley JC, Villacide JM, van Nouhuys S. Patch time allocation by a parasitoid: the influence of con-specifics, host abundance and distance to the patch. J Insect Behav. 2010;23:431–40.

[55] Fischbein D, Bettinelli J, Bernstein C, Corley JC. Patch choice from a distance and use of habitat information during foraging by the parasitoid *Ibalia leucospoides*. Ecol Entomol. 2012;37:161–8.

