# Supplementary material for: Suitability of eastern pines for oviposition and survival of Sirex noctilio F
Source: PLoS One. 2017 Mar 23;12(3):e0174532. doi: 10.1371/journal.pone.0174532 (PMC5363988; doi:10.1371/journal.pone.0174532)
Supplement: S1 Table — Data includes number of Sirex noctilio eggs oviposited by females in the parent generation (estimate; see text for details), and number of adult S. noctilio in the F2 generation. (DOCX) [file pone.0174532.s001.docx]

**Supplementary Table 1. Data collected from each log sample.** Data includes number of *Sirex noctilio* eggs oviposited by females in the parent generation (estimate; see text for details), and number of adult S. noctilio in the F2 generation.

| Pine species | Tree number | Treatment | No. eggs | No. adults |
| --- | --- | --- | --- | --- |
| Jack | 1 | Cage | 45 | 0 |
| Jack | 1 | No cage | 1 | 0 |
| Jack | 2 | Cage | 0 | 0 |
| Jack | 2 | No cage | 21 | 0 |
| Jack | 3 | Cage | 76 | 1 |
| Jack | 3 | No cage | 15 | 0 |
| Jack | 4 | Cage | 17 | 2 |
| Jack | 4 | No cage | 102 | 2 |
| Jack | 5 | Cage | 0 | 0 |
| Jack | 5 | No cage | 0 | 0 |
| Jack | 6 | Cage | 16 | 0 |
| Jack | 6 | No cage | 18 | 0 |
| Jack | 7 | Cage | 49 | 0 |
| Jack | 7 | No cage | 19 | 0 |
| Jack | 8 | Cage | 102 | 0 |
| Jack | 8 | No cage | 125 | 1 |
| Jack | 9 | Cage | 70 | 0 |
| Jack | 9 | No cage | 36 | 0 |
| Jack | 10 | Cage | 1 | 0 |
| Jack | 10 | No cage | 18 | 0 |
| Red | 1 | Cage | 79 | 8 |
| Red | 1 | No cage | 12 | 0 |
| Red | 2 | Cage | 107 | 17 |
| Red | 2 | No cage | 63 | 2 |
| Red | 3 | Cage | 72 | 0 |
| Red | 3 | No cage | 57 | 0 |
| Red | 4 | Cage | 14 | 0 |
| Red | 4 | No cage | 80 | 1 |
| Red | 5 | Cage | 0 | 0 |
| Red | 5 | No cage | 5 | 0 |
| Red | 6 | Cage | 6 | 0 |
| Red | 6 | No cage | 4 | 0 |
| Red | 7 | Cage | 0 | 0 |
| Red | 7 | No cage | 59 | 11 |
| Red | 8 | Cage | 30 | 7 |
| Red | 8 | No cage | 40 | 4 |
| Red | 9 | Cage | 69 | 3 |
| Red | 9 | No cage | 12 | 0 |
| Red | 10 | Cage | 0 | 0 |
| Red | 10 | No cage | 0 | 0 |
| Scots | 1 | Cage | 25 | 0 |
| Scots | 1 | No cage | 2 | 0 |
| Scots | 2 | Cage | 57 | 4 |
| Scots | 2 | No cage | 84 | 4 |
| Scots | 3 | Cage | 33 | 1 |
| Scots | 3 | No cage | 69 | 0 |
| Scots | 4 | Cage | 9 | 0 |
| Scots | 4 | No cage | 0 | 0 |
| Scots | 5 | Cage | 28 | 0 |
| Scots | 5 | No cage | 50 | 3 |
| Scots | 6 | Cage | 1 | 0 |
| Scots | 6 | No cage | 47 | 0 |
| Scots | 7 | Cage | 38 | 1 |
| Scots | 7 | No cage | 86 | 2 |
| Scots | 8 | Cage | 59 | 16 |
| Scots | 8 | No cage | 152 | 13 |
| Scots | 9 | Cage | 160 | 14 |
| Scots | 9 | No cage | 58 | 5 |
| Scots | 10 | Cage | 33 | 0 |
| Scots | 10 | No cage | 120 | 1 |
| White | 1 | Cage | 0 | 0 |
| White | 1 | No cage | 0 | 0 |
| White | 2 | Cage | 11 | 5 |
| White | 2 | No cage | 21 | 4 |
| White | 3 | Cage | 3 | 0 |
| White | 3 | No cage | 0 | 0 |
| White | 4 | Cage | 0 | 0 |
| White | 4 | No cage | 0 | 0 |
| White | 5 | Cage | 3 | 2 |
| White | 5 | No cage | 1 | 0 |
| White | 6 | Cage | 25 | 7 |
| White | 6 | No cage | 18 | 1 |
| White | 7 | Cage | 0 | 0 |
| White | 7 | No cage | 0 | 0 |
| White | 8 | Cage | 1 | 0 |
| White | 8 | No cage | 21 | 3 |
| White | 9 | Cage | 5 | 0 |
| White | 9 | No cage | 0 | 0 |
| White | 10 | Cage | 0 | 0 |
| White | 10 | No cage | 0 | 0 |
